# Crystal structures and Hirshfeld surface analyses of di­phenyl­methyl 2-(3,5-di­meth­oxy­phen­yl)acetate and di­phenyl­methyl 2-(3,4,5-tri­meth­oxy­phen­yl)acetate

**DOI:** 10.1107/S2056989025004943

**Published:** 2025-06-12

**Authors:** Manivel Kavitha, Chandiran Jayakodi, Ganesan Meenambigai, Sekar Janarthanan, Srinivasan Pazhamalai, Sivashanmugam Selvanayagam

**Affiliations:** a Department of Chemistry Annamalai University, Annamalainagar Chidambaram 608 002 India; b PG & Research Department of Physics, Government Arts College, Melur 625 106, India; Vienna University of Technology, Austria

**Keywords:** crystal structure, acetate derivatives, superposition, C—H⋯O intra and inter­molecular hydrogen bonds, conformational differences

## Abstract

The title compounds, C_23_H_22_O_4_, (I), and C_24_H_24_O_5_, (II), differ in the presence of a meth­oxy group instead of a hydrogen atom between two meth­oxy groups attached to the phenyl ring of the phenyl acetate moiety, which affects not only the symmetry and number of formula units [triclinic, *P*1, *Z* = 2 for (I); monoclinic, *P*2_1_/*n*, *Z* = 4 for (II)], but also the mol­ecular conformations. An overlay of the two mol­ecular structures reveals a large root-mean-square-deviation of 2.4 Å. Intra and inter­molecular C—H⋯O hydrogen bonds are responsible for the consolidation of the mol­ecular conformations and the crystal packing of both structures. Their inter­molecular inter­actions were qu­anti­fied and analysed using Hirshfeld surface analysis, revealing that H⋯H inter­actions contribute most to the crystal packing.

## Abstract 2

The title compounds, C_23_H_22_O_4_, (I), and C_24_H_24_O_5_, (II), differ in the presence of a meth­oxy atom instead of a hydrogen atom between two meth­oxy groups at the phenyl ring, which greatly affects the mol­ecular conformations and the symmetries of the crystals.

## 1. Chemical context

Esters are fundamental synthons or synthetic targets, as they are widely found in bioactive natural compounds and thus are important in both pharmaceutical and industrial applications. Esterifications are typically carried out under mild conditions, making them suitable for the synthesis of sensitive and labile compounds (Chiodi & Ishihara, 2024 ▸).
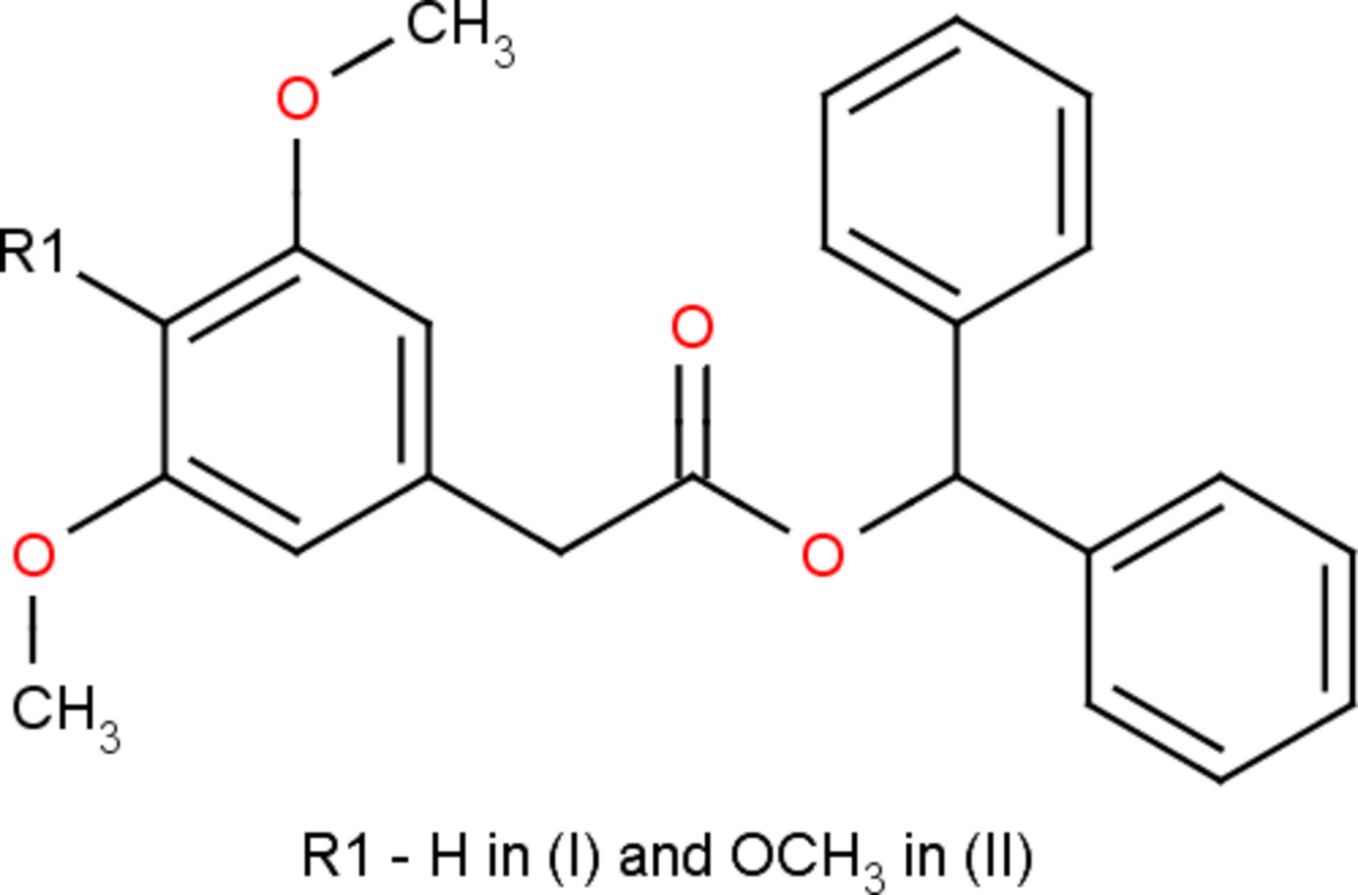


In the present work, the synthesis, structural and Hirshfeld surface analysis of the esters di­phenyl­methyl 2-(3,5-di­meth­oxy­phen­yl)acetate, (I), and di­phenyl­methyl-2-(3,4,5-tri­meth­oxy­phen­yl)acetate, (II), are reported.

## 2. Structural commentary

**Figure 1:**
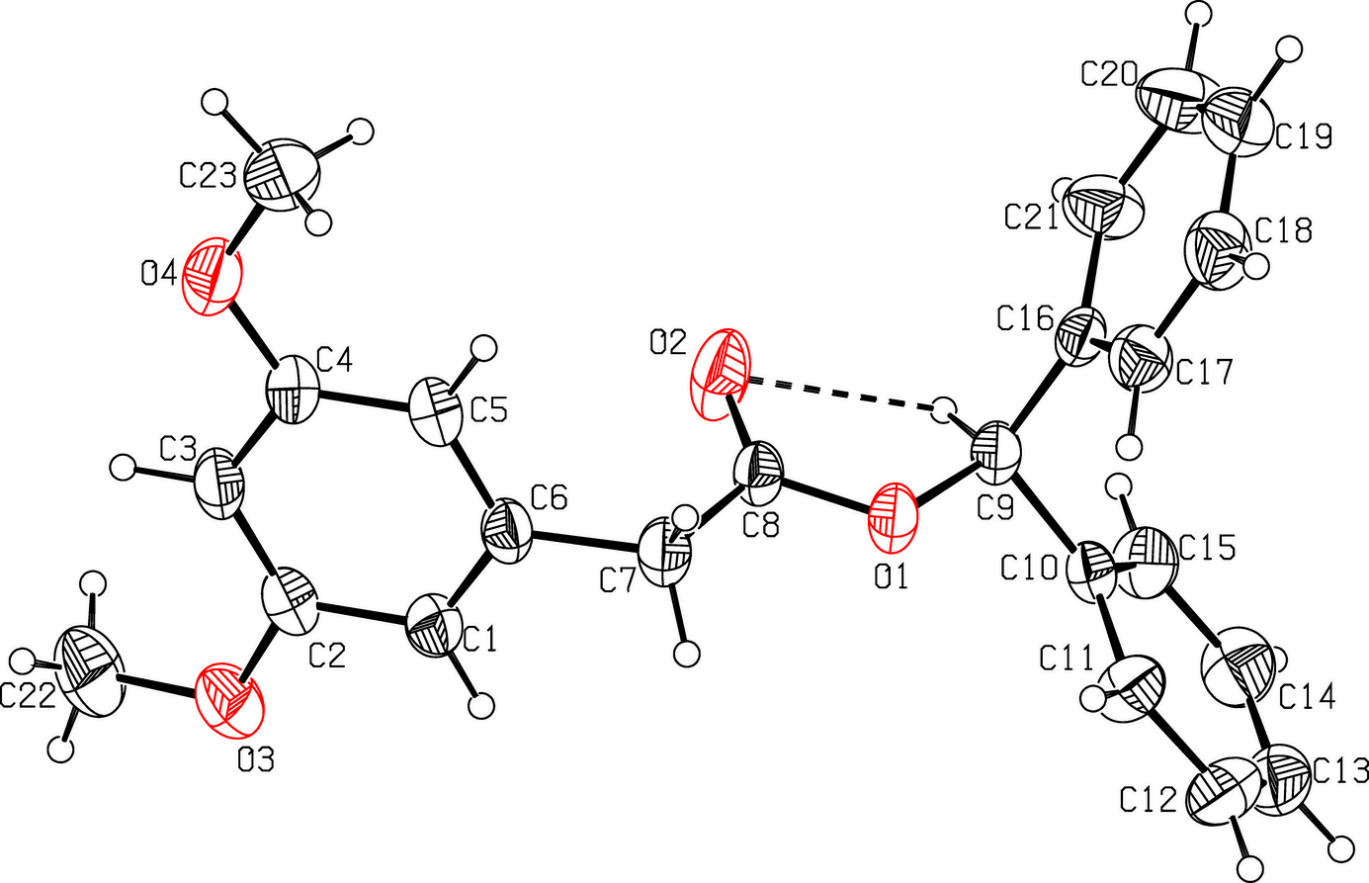
The mol­ecular structure of compound (I) with displacement ellipsoids drawn at the 30% probability level. The intra­molecular C—H⋯O inter­action is shown as a dashed line.

**Figure 3:**
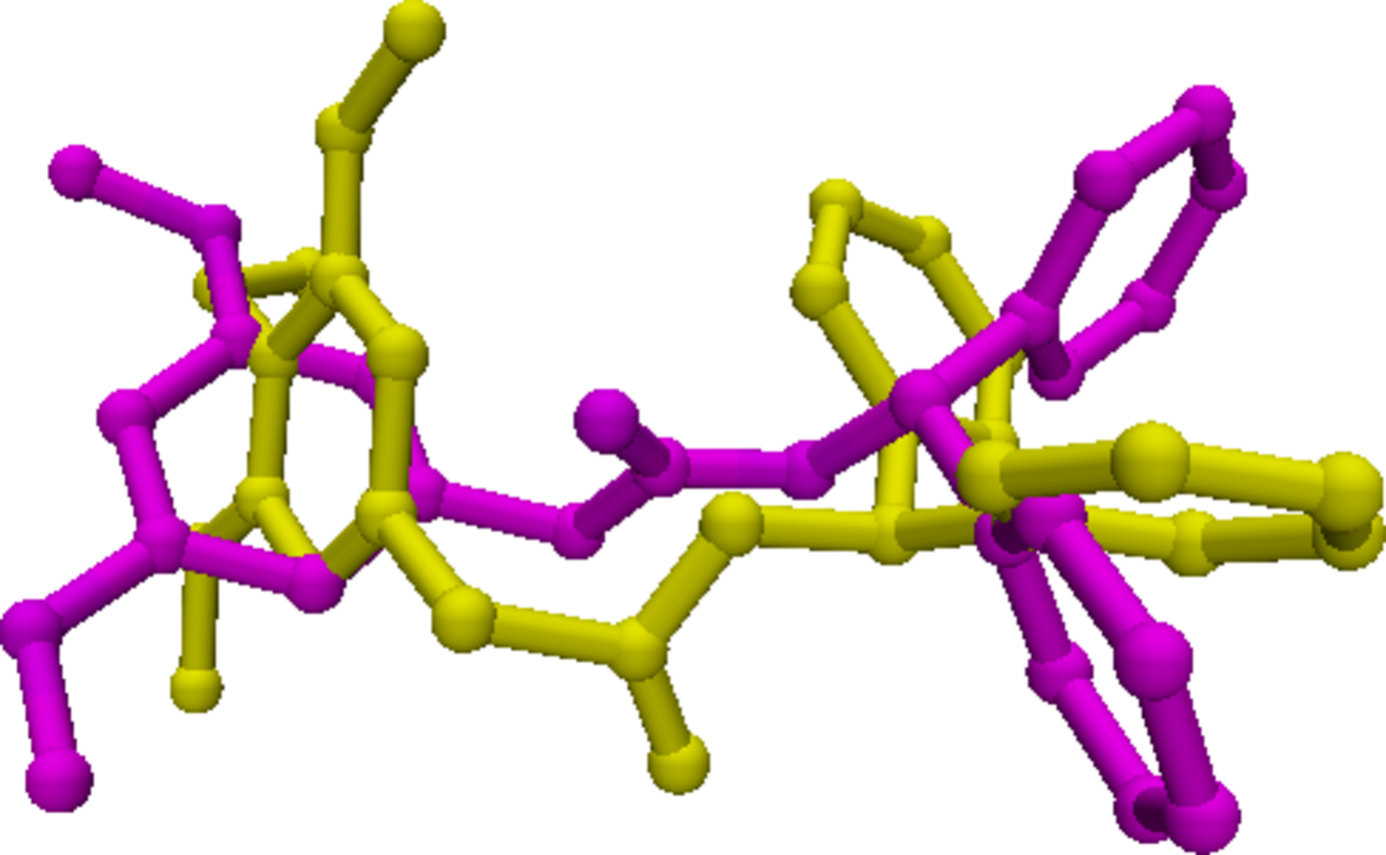
Superposition of mol­ecules (I) (violet) and (II) (yellow), except for the meth­oxy group [O5–C24 in (II)]. The overlay plot was produced with *Qmol* (Gans & Shalloway, 2001 ▸).

The mol­ecular structures of (I) and (II) are illustrated in Figs. 1 ▸ and 2 ▸. Although the two mol­ecules differ only in the presence of a meth­oxy group instead of a hydrogen atom in between two meth­oxy groups, they adopt different conformations, as an overlay plot of the two mol­ecules shows (Fig. 3 ▸); the root-mean-square-deviation is 2.4 Å. The dimeth­oxy phenyl ring in (I) is planar with a maximum deviation of −0.005 (3) Å for atom C1 and its attached atoms of the meth­oxy groups (O3, C22; O4, C23) deviate by 0.015 (2), 0.228 (4), −0.020 (2) and 0.005 (4) Å, respectively, from this plane. The acetate moiety (C7/C8/O1/C9/O2) in (I) is nearly planar with a maximum deviation of −0.110 (2) Å for atom O2 from the best plane. This moiety forms a dihedral angle of 83.7 (1)° with respect to the dimeth­oxy phenyl ring. The two phenyl rings (C10–C15; C16–C21) of the di­phenyl­methyl moiety in (I) are oriented at a dihedral angle of 71.1 (2)°.

The trimeth­oxy phenyl ring in (II) is planar with a maximum deviation of 0.007 (2) Å for atom C1 and its attached meth­oxy atoms (O3, C22; O4, C23; O5 C24) deviate by 0.015 (2), 0.038 (3), 0.031 (2), −0.067 (3), 0.076 (2) and −1.165 (5) Å, respectively, from this plane. The acetate moiety (C7/C8/O1/C9/O2) in (II) is planar with a maximum deviation of 0.005 (2) Å for atom C9. This moiety forms a dihedral angle of 71.4 (1)° with respect to the trimeth­oxy phenyl ring. The two phenyl rings (C10–C15; C16–C21) in (II) are oriented at a dihedral angle of 65.6 (2)°.

Weak intra­molecular C—H⋯O hydrogen bonds between the methine H atom of the di­phenyl­methyl entity consolidate the mol­ecular conformation in both cases (Figs. 1 ▸, 2 ▸; Tables 1 ▸, 2 ▸).

## 3. Supra­molecular features

**Figure 4:**
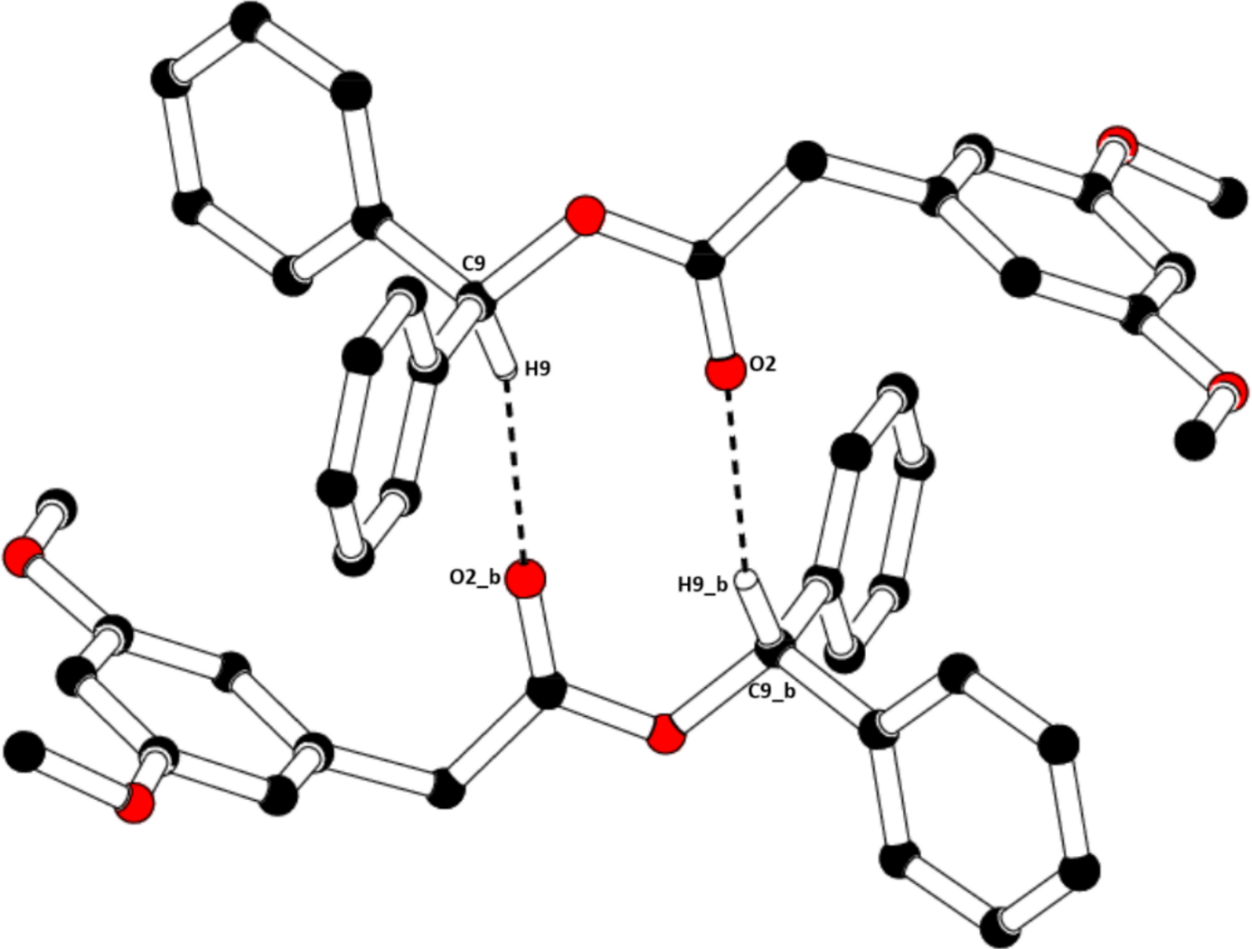
The formation of a centrosymmetric dimer in the crystal structure of (I) through C—H⋯O hydrogen bonds (dashed lines). [Symmetry code: (*b*) −*x*, −*y* + 2, −*z* + 1]

**Figure 5:**
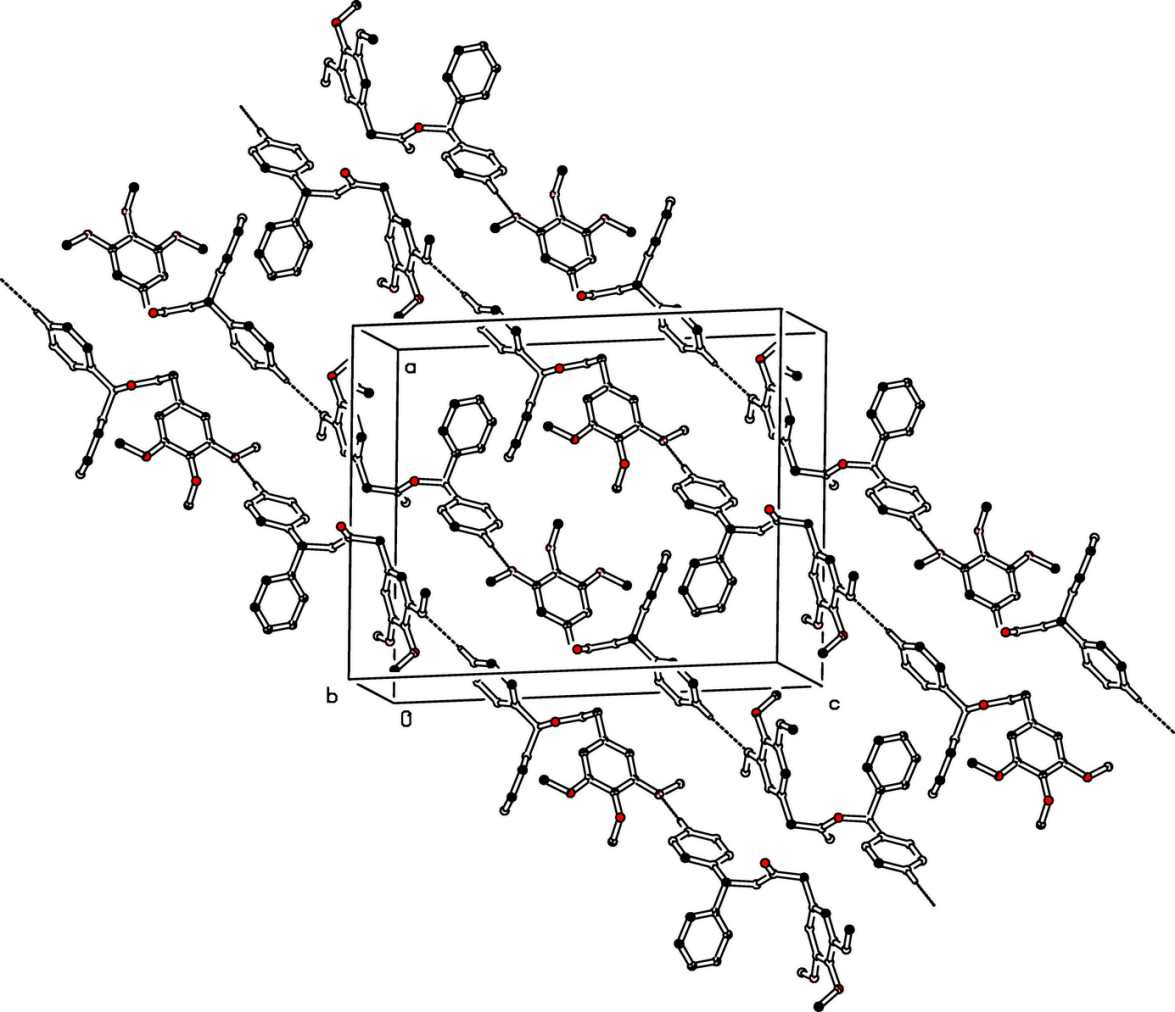
The crystal packing of (II) viewed approximately down the *b* axis. C—H⋯O inter­molecular hydrogen bonds are shown as dashed lines; for clarity H atoms not involved in these hydrogen bonds have been omitted.

In the crystal of (I), mol­ecules associate pairwise *via* C9—H9⋯O2^i^ hydrogen bonds (Table 1 ▸) into inversion dimers with an 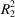 (10) graph-set motif (Etter *et al.*, 1990 ▸), as shown in Fig. 4 ▸. In the crystal of (II), mol­ecules associate into a *C*(13) chain by C19—H19⋯O4^i^ hydrogen bonds running in anti-parallel manner along [101] (Table 2 ▸; Fig. 5 ▸).

## 4. Hirshfeld surface analysis

**Figure 6:**
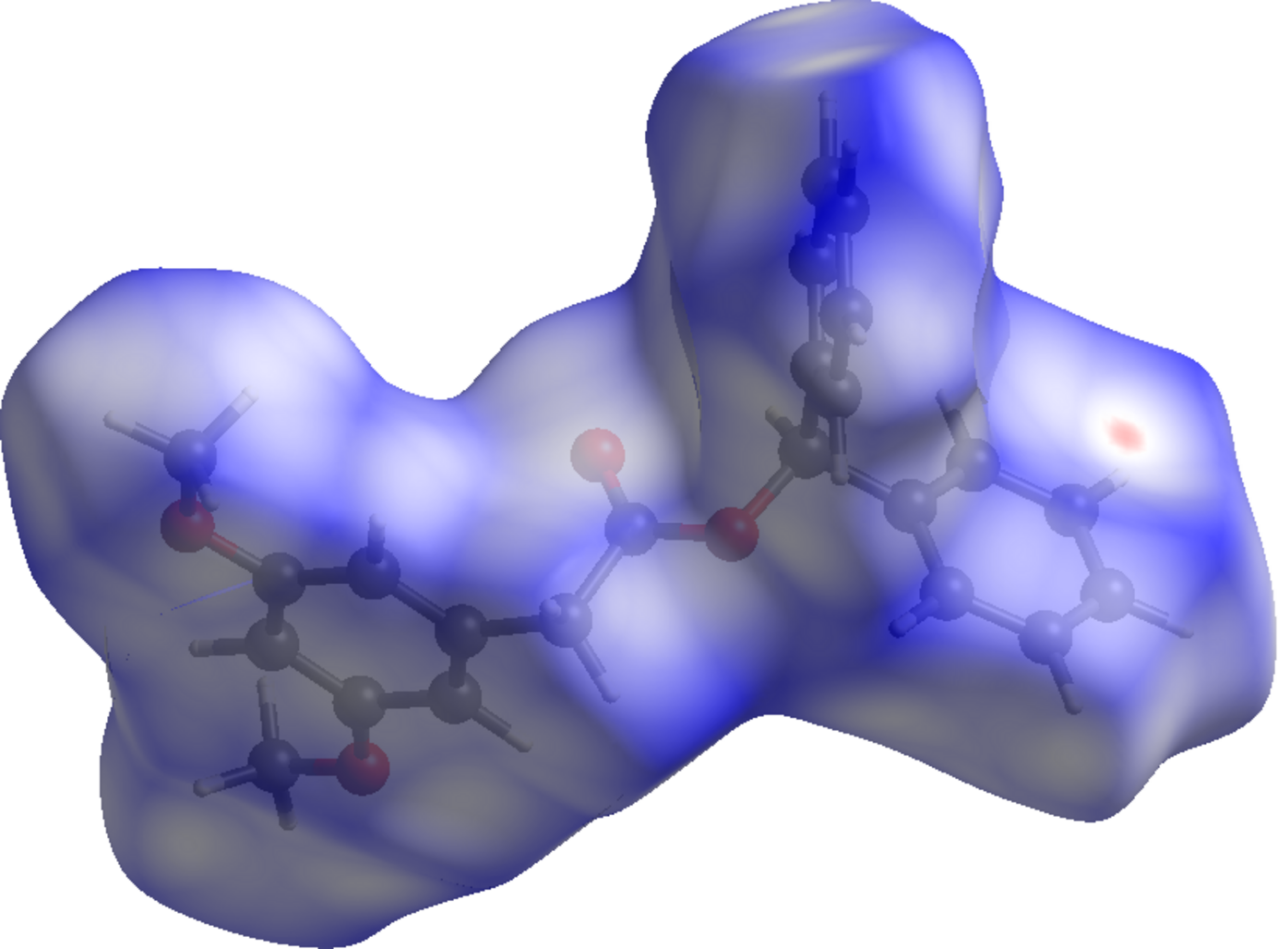
A view of the Hirshfeld surface mapped over *d*_norm_ for (I).

To further characterize the inter­molecular inter­actions in the title compound, a Hirshfeld surface (HS) analysis (Spackman & Jayatilaka, 2009 ▸) was carried out with *CrystalExplorer* (Spackman *et al.*, 2021 ▸). The HS mapped over *d*_norm_ for (I) and (II) are illustrated in Figs. 6 ▸ and 7 ▸, respectively, with a colour scheme to indicate contacts shorter (red areas), equal to (white areas), or longer than (blue areas) the sum of the van der Waals radii (Ashfaq *et al.*, 2021 ▸).

**Figure 8:**
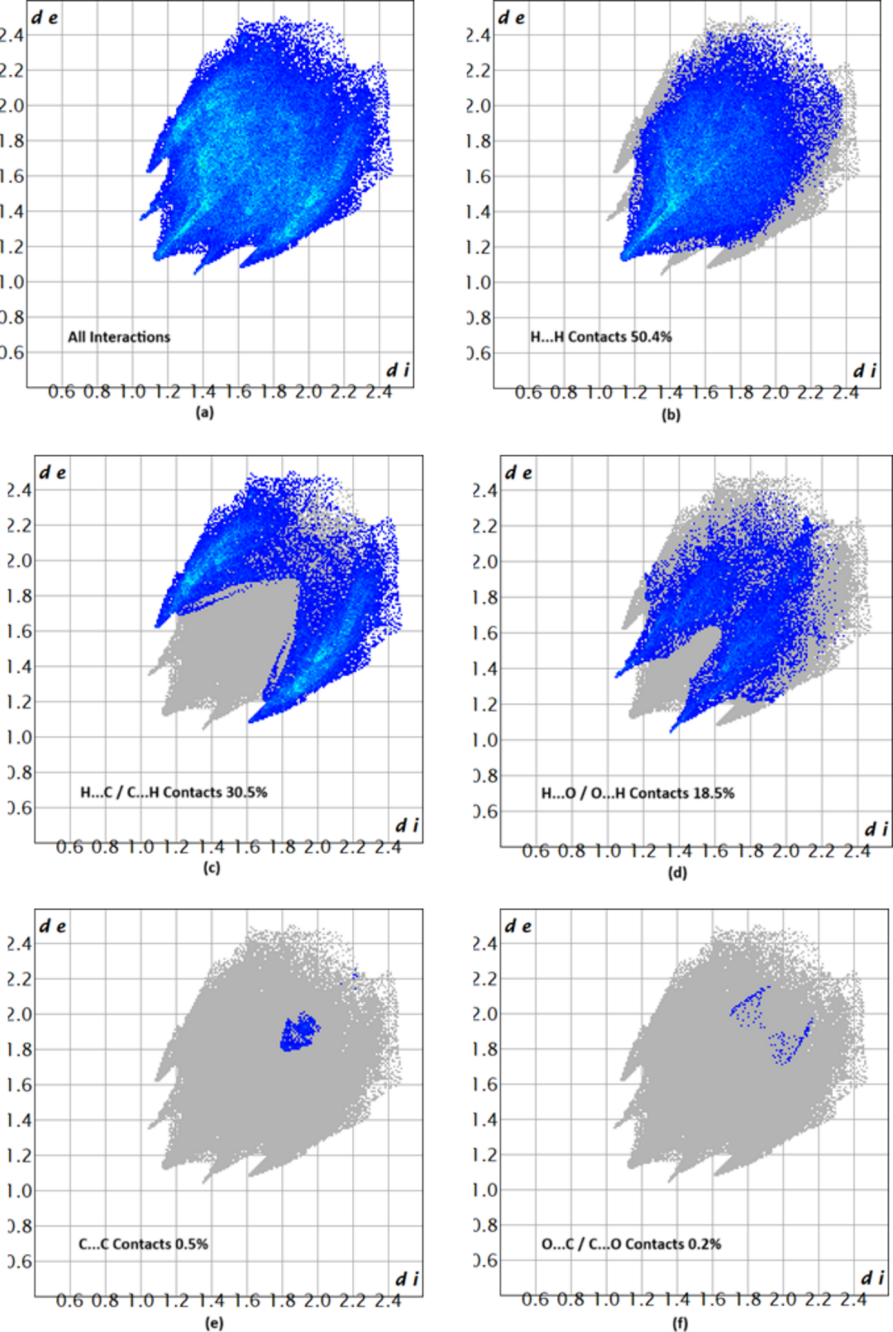
Two-dimensional fingerprint plots for (I), showing (*a*) all inter­actions, and delineated into (*b*) H⋯H, (*c*) H⋯C/C⋯H, (*d*) H⋯O/O⋯H, (*e*) C⋯C and (*f*) O⋯C/C⋯O inter­actions. The *d*_i_ and *d*_e_ values are the closest inter­nal and external distances (in Å) from given points on the Hirshfeld surface.

**Figure 9:**
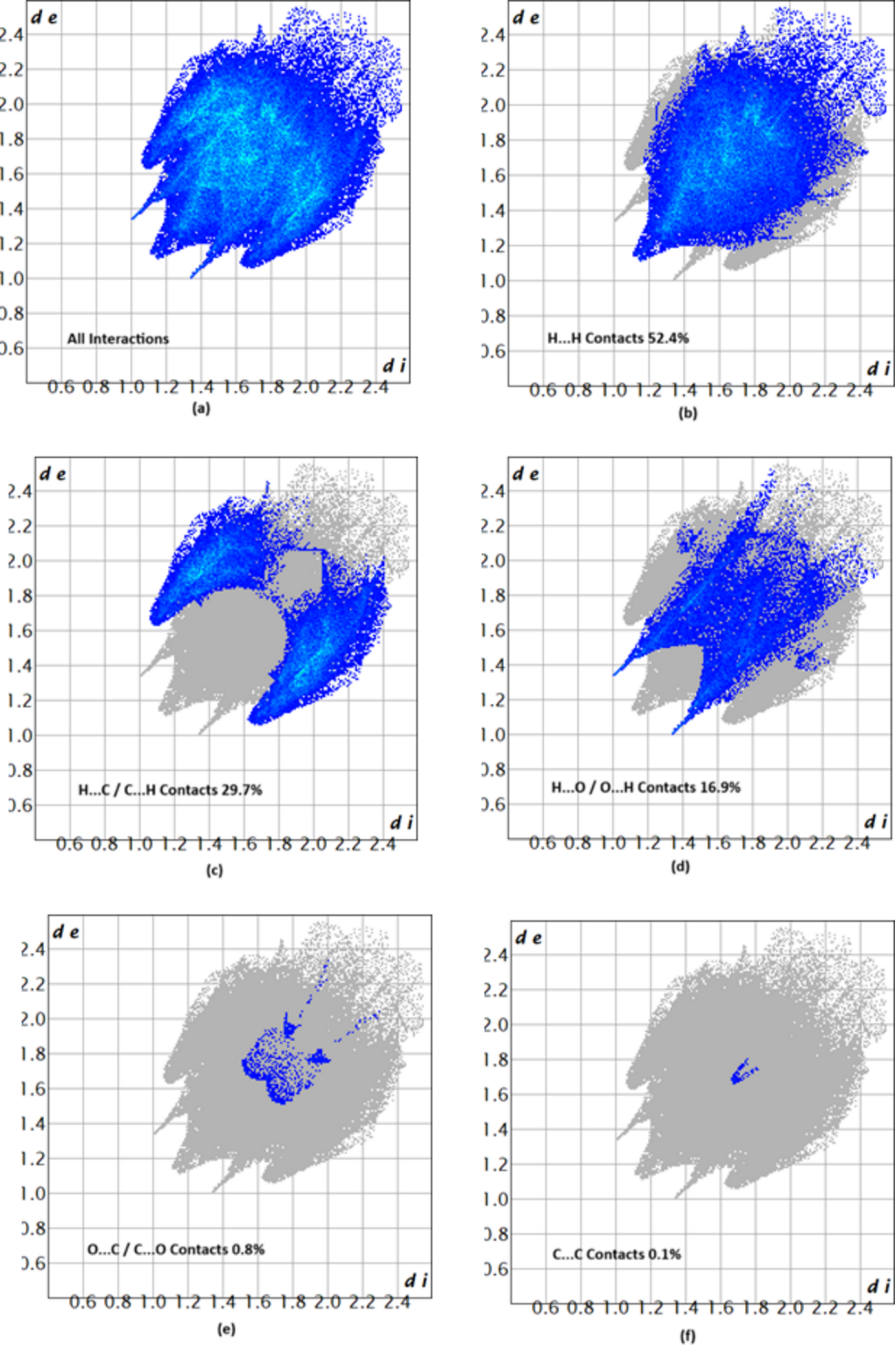
Two-dimensional fingerprint plots for compound (II), showing (*a*) all inter­actions, and delineated into (*b*) H⋯H, (*c*) H⋯C/C⋯H, (*d*) H⋯O/O⋯H, (*e*) O⋯C/C⋯O and (*f*) C⋯C inter­actions.

The associated two-dimensional fingerprint plots (McKinnon *et al.*, 2007 ▸) provide qu­anti­tative information about the non-covalent inter­actions in the crystal packing in terms of the percentage contribution of the inter­atomic contacts (Spackman & McKinnon, 2002 ▸). The overall two-dimensional fingerprint plot for compound (I) is shown in Fig. 8 ▸*a*. H⋯H and H⋯C/C⋯H contacts are the main contributors to the crystal packing, followed by H⋯O/O⋯H, C⋯C and O⋯C/C⋯O contacts for compound (I), as shown in Fig. 8 ▸*b*–*f*. In compound (II), the overall two-dimensional fingerprint is shown in Fig. 9 ▸*a*. Again, H⋯H and H⋯C/C⋯H contacts are the main contributors to the crystal packing, followed by H⋯O/O⋯H, O⋯C/C⋯O and C⋯C contacts (Fig. 9 ▸*b*–*f*). The HS analysis confirms the importance of H-atom contacts in establishing the packing (Hathwar *et al.*, 2015 ▸).

## 5. Synthesis and crystallization

For the synthesis of (I), a mixture containing 3,5-di­meth­oxy­phenyl­acetic acid (0.1 mmol), benzhydrol (0.1 mmol), *N*,*N*′-di­cyclo­hexyl­carbodi­imide (0.4 g), and 4-di­methyl­amino­pyridine (0.8 g) was placed into a 250 ml round-bottom flask. To this, 100 ml of di­chloro­methane were added, and the reaction mixture was refluxed on a water bath at 321 K for 9–11 h. After completion of the reaction, as monitored by thin-layer chromatography (TLC), the precipitate formed was filtered off, and the solvent was evaporated to dryness. The crude product was then purified by column chromatography using a solvent system of ethyl acetate and petroleum ether in a 1:4 (*v*:*v*) ratio. The separated product was dried *in vacuo*, giving colourless crystals with 85% yield.

For the synthesis of (II), a mixture containing 3,4,5-di­meth­oxy­phenyl­acetic acid (0.1 mmol), benzhydrol (0.1 mmol), *N*,*N*′-di­cyclo­hexyl­carbodi­imide (0.4 g), and 4-di­methyl­amino­pyridine (0.8 g) was placed into a 250 ml round-bottom flask. To this, 100 ml of di­chloro­methane was added, and the reaction mixture was refluxed on a water bath at 321 K for 9–11 h. Upon completion of the reaction, as monitored by thin-layer chromatography (TLC), the precipitate formed was filtered off, and the solvent was evaporated to dryness. The crude product was then purified by column chromatography using a solvent system of ethyl acetate and petroleum ether in a 1:4 (*v*:*v*) ratio. The resulting compound was obtained as colourless crystals with a 90% yield.

For (I) and (II), the solid products were recrystallized from methanol to obtain crystals suitable for X-ray analysis.

## 6. Refinement

Crystal data, data collection and structure refinement details are summarized in Table 3 ▸. In both (I) and (II), H atoms were placed in idealized positions and allowed to ride on their parent atoms: C—H = 0.93–0.98 Å, with *U*_iso_(H) = 1.5*U*_eq_(C-meth­yl) and 1.2*U*_eq_(C) for other H atoms.

## Supplementary Material

Crystal structure: contains datablock(s) I, II, global. DOI: 10.1107/S2056989025004943/wm5759sup1.cif

Structure factors: contains datablock(s) I. DOI: 10.1107/S2056989025004943/wm5759Isup2.hkl

Structure factors: contains datablock(s) II. DOI: 10.1107/S2056989025004943/wm5759IIsup3.hkl

Supporting information file. DOI: 10.1107/S2056989025004943/wm5759Isup4.cml

Supporting information file. DOI: 10.1107/S2056989025004943/wm5759IIsup5.cml

CCDC references: 2400579, 2400580

Additional supporting information:  crystallographic information; 3D view; checkCIF report

## Figures and Tables

**Figure 2:**
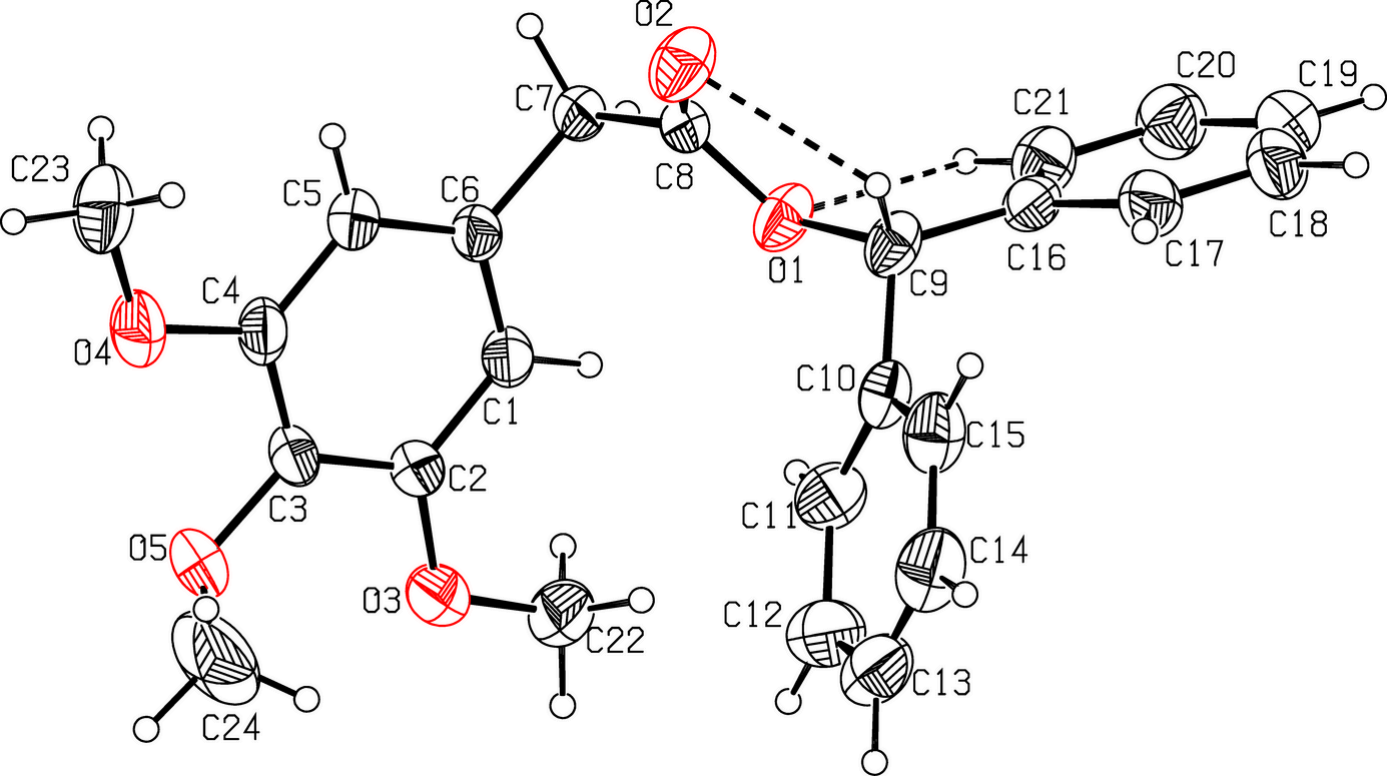
The mol­ecular structure of compound (II) with displacement ellipsoids drawn at the 30% probability level. Intra­molecular C—H⋯O inter­actions are shown as dashed lines.

**Figure 7:**
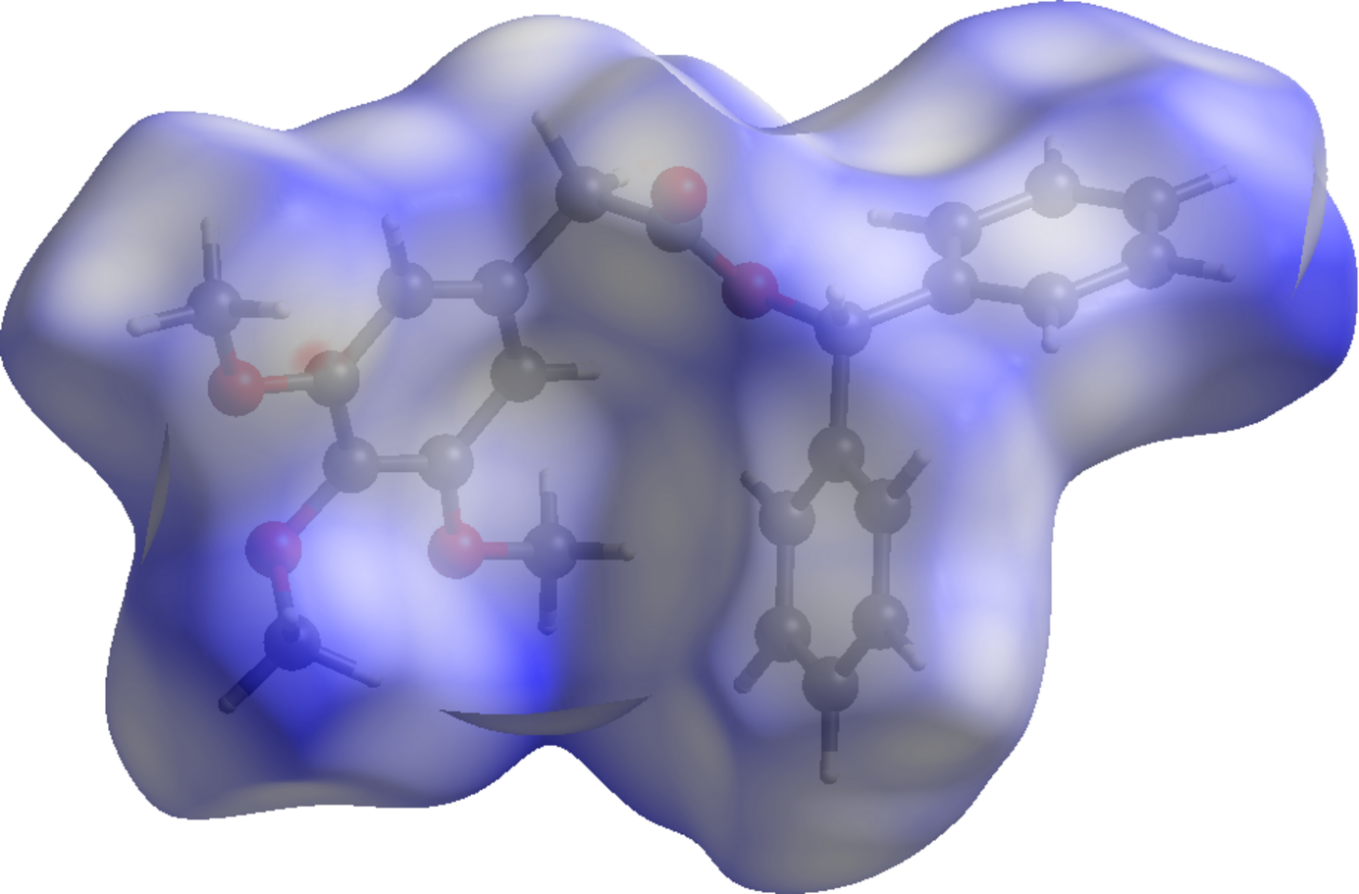
A view of the Hirshfeld surface mapped over *d*_norm_ for (II).

**Table 1:** Hydrogen-bond geometry (Å, °) for (I)

*D*—H⋯*A*	*D*—H	H⋯*A*	*D*⋯*A*	*D*—H⋯*A*
C9—H9⋯O2	0.98	2.30	2.667 (3)	101
C9—H9⋯O2^i^	0.98	2.50	3.444 (3)	162

Symmetry code: (i) 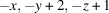.

**Table 2:** Hydrogen-bond geometry (Å, °) for (II)

*D*—H⋯*A*	*D*—H	H⋯*A*	*D*⋯*A*	*D*—H⋯*A*
C9—H9⋯O2	0.98	2.24	2.691 (3)	107
C21—H21⋯O1	0.93	2.39	2.740 (3)	102
C19—H19⋯O4^i^	0.93	2.50	3.427 (3)	175

Symmetry code: (i) 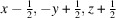.

**Table 3:** Experimental details

	(I)	(II)
Crystal data		
Chemical formula	C_23_H_22_O_4_	C_24_H_24_O_5_
*M*_r_	362.40	392.43
Crystal system, space group	Triclinic, *P* 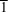	Monoclinic, *P*2_1_/*n*
Temperature (K)	300	300
*a*, *b*, *c* (Å)	8.5327 (12), 11.0216 (15), 11.4369 (16)	17.2290 (14), 5.5037 (5), 22.1140 (18)
α, β, γ (°)	111.817 (4), 96.977 (4), 99.925 (4)	90, 92.256 (2), 90
*V* (Å^3^)	963.1 (2)	2095.3 (3)
*Z*	2	4
Radiation type	Mo *K*α	Mo *K*α
μ (mm^−1^)	0.09	0.09
Crystal size (mm)	0.19 × 0.18 × 0.17	0.28 × 0.09 × 0.07

Data collection		
Diffractometer	Bruker APEXII CCD	Bruker APEXII CCD
Absorption correction	Multi-scan (*SADABS*; Krause *et al.*, 2015 ▸)	Multi-scan (*SADABS*; Krause *et al.*, 2015 ▸)
*T*_min_, *T*_max_	0.674, 0.746	0.694, 0.746
No. of measured, independent and observed [*I* > 2σ(*I*)] reflections	24070, 4782, 2717	39211, 5016, 2516
*R*_int_	0.047	0.074
(sin θ/λ)_max_ (Å^−1^)	0.668	0.660

Refinement		
*R*[*F*^2^ > 2σ(*F*^2^)], *wR*(*F*^2^), *S*	0.065, 0.202, 1.04	0.057, 0.176, 1.00
No. of reflections	4782	5016
No. of parameters	245	263
H-atom treatment	H-atom parameters constrained	H-atom parameters constrained
Δρ_max_, Δρ_min_ (e Å^−3^)	0.68, −0.45	0.25, −0.18

Computer programs: *APEX3* and *SAINT* (Bruker, 2017 ▸), *SHELXT* (Sheldrick, 2015*a* ▸), *ORTEP-3 for Windows* (Farrugia, 2012 ▸), *PLATON* (Spek, 2020 ▸) and *SHELXL* (Sheldrick, 2015*b* ▸).

## supplementary crystallographic information

### Diphenylmethyl 2-(3,5-dimethoxyphenyl)acetate (I) Crystal data

**Table:**

C_23_H_22_O_4_	*Z* = 2
*M**_r_* = 362.40	*F*(000) = 384
Triclinic, *P*1	*D*_x_ = 1.250 Mg m^−^^3^
*a* = 8.5327 (12) Å	Mo *K*α radiation, λ = 0.71073 Å
*b* = 11.0216 (15) Å	Cell parameters from 6939 reflections
*c* = 11.4369 (16) Å	θ = 2.5–24.7°
α = 111.817 (4)°	µ = 0.09 mm^−^^1^
β = 96.977 (4)°	*T* = 300 K
γ = 99.925 (4)°	Block, colourless
*V* = 963.1 (2) Å^3^	0.19 × 0.18 × 0.17 mm

### Diphenylmethyl 2-(3,5-dimethoxyphenyl)acetate (I) Data collection

**Table:**

Bruker APEXII CCD diffractometer	2717 reflections with *I* > 2σ(*I*)
Radiation source: i-mu-s microfocus source	*R*_int_ = 0.047
φ and ω scans	θ_max_ = 28.3°, θ_min_ = 2.0°
Absorption correction: multi-scan (SADABS; Krause *et al.*, 2015)	*h* = −11→11
*T*_min_ = 0.674, *T*_max_ = 0.746	*k* = −14→14
24070 measured reflections	*l* = −15→15
4782 independent reflections	

### Diphenylmethyl 2-(3,5-dimethoxyphenyl)acetate (I) Refinement

**Table:**

Refinement on *F*^2^	Hydrogen site location: inferred from neighbouring sites
Least-squares matrix: full	H-atom parameters constrained
*R*[*F*^2^ > 2σ(*F*^2^)] = 0.065	*w* = 1/[σ^2^(*F*_o_^2^) + (0.0762*P*)^2^ + 0.4138*P*] where *P* = (*F*_o_^2^ + 2*F*_c_^2^)/3
*wR*(*F*^2^) = 0.202	(Δ/σ)_max_ < 0.001
*S* = 1.04	Δρ_max_ = 0.68 e Å^−^^3^
4782 reflections	Δρ_min_ = −0.45 e Å^−^^3^
245 parameters	Extinction correction: SHELXL (Sheldrick, 2015b), Fc^*^=kFc[1+0.001xFc^2^λ^3^/sin(2θ)]^-1/4^
0 restraints	Extinction coefficient: 0.060 (11)

### Diphenylmethyl 2-(3,5-dimethoxyphenyl)acetate (I) Special details

**Table:**

Geometry. All esds (except the esd in the dihedral angle between two l.s. planes) are estimated using the full covariance matrix. The cell esds are taken into account individually in the estimation of esds in distances, angles and torsion angles; correlations between esds in cell parameters are only used when they are defined by crystal symmetry. An approximate (isotropic) treatment of cell esds is used for estimating esds involving l.s. planes.

### Diphenylmethyl 2-(3,5-dimethoxyphenyl)acetate (I) Fractional atomic coordinates and isotropic or equivalent isotropic displacement parameters (Å^2^)

**Table:**

	*x*	*y*	*z*	*U*_iso_*/*U*_eq_	
O1	0.06071 (19)	0.86788 (15)	0.68377 (15)	0.0598 (5)	
O2	−0.1148 (3)	0.8780 (2)	0.5309 (2)	0.0948 (8)	
O3	−0.3040 (3)	0.37482 (19)	0.13567 (18)	0.0835 (6)	
O4	−0.7070 (2)	0.5897 (2)	0.31738 (19)	0.0879 (7)	
C1	−0.2464 (3)	0.5223 (2)	0.3518 (2)	0.0593 (6)	
H1	−0.143764	0.504369	0.357291	0.071*	
C2	−0.3584 (3)	0.4594 (2)	0.2359 (2)	0.0606 (6)	
C3	−0.5109 (3)	0.4842 (2)	0.2268 (2)	0.0630 (7)	
H3	−0.585815	0.442242	0.149246	0.076*	
C4	−0.5514 (3)	0.5729 (2)	0.3353 (2)	0.0616 (6)	
C5	−0.4413 (3)	0.6365 (2)	0.4506 (2)	0.0592 (6)	
H5	−0.469808	0.695759	0.522359	0.071*	
C6	−0.2869 (3)	0.6112 (2)	0.4585 (2)	0.0536 (6)	
C7	−0.1637 (3)	0.6853 (2)	0.5823 (2)	0.0599 (6)	
H7A	−0.218876	0.699926	0.654003	0.072*	
H7B	−0.088345	0.630395	0.588522	0.072*	
C8	−0.0708 (3)	0.8180 (2)	0.59202 (19)	0.0504 (5)	
C9	0.1649 (3)	0.9959 (2)	0.7017 (2)	0.0504 (5)	
H9	0.143304	1.010726	0.622524	0.060*	
C10	0.3389 (3)	0.9848 (2)	0.72620 (19)	0.0503 (5)	
C11	0.3878 (3)	0.9112 (3)	0.7918 (3)	0.0698 (7)	
H11	0.311265	0.862025	0.818700	0.084*	
C12	0.5495 (4)	0.9096 (3)	0.8181 (3)	0.0836 (9)	
H12	0.581182	0.860409	0.863666	0.100*	
C13	0.6628 (4)	0.9794 (3)	0.7781 (3)	0.0797 (8)	
H13	0.771632	0.978449	0.796676	0.096*	
C14	0.6161 (4)	1.0504 (3)	0.7110 (3)	0.0831 (9)	
H14	0.693159	1.097307	0.682559	0.100*	
C15	0.4544 (3)	1.0536 (3)	0.6845 (3)	0.0683 (7)	
H15	0.423666	1.102371	0.638211	0.082*	
C16	0.1289 (2)	1.1093 (2)	0.8108 (2)	0.0495 (5)	
C17	0.1159 (3)	1.1014 (3)	0.9267 (2)	0.0660 (7)	
H17	0.129286	1.024538	0.938563	0.079*	
C18	0.0833 (4)	1.2066 (3)	1.0257 (3)	0.0804 (8)	
H18	0.072381	1.199310	1.102884	0.096*	
C19	0.0673 (4)	1.3205 (3)	1.0099 (3)	0.0890 (9)	
H19	0.044447	1.391048	1.075939	0.107*	
C20	0.0848 (5)	1.3307 (3)	0.8974 (4)	0.1044 (11)	
H20	0.076628	1.409502	0.887418	0.125*	
C21	0.1145 (4)	1.2251 (3)	0.7976 (3)	0.0816 (8)	
H21	0.124898	1.233051	0.720604	0.098*	
C22	−0.4026 (5)	0.3230 (4)	0.0098 (3)	0.1043 (11)	
H22A	−0.350462	0.265698	−0.050669	0.156*	
H22B	−0.506572	0.272258	0.008731	0.156*	
H22C	−0.417052	0.396179	−0.013418	0.156*	
C23	−0.7547 (4)	0.6812 (3)	0.4234 (3)	0.0870 (9)	
H23A	−0.865185	0.683867	0.398647	0.130*	
H23B	−0.745738	0.652439	0.493080	0.130*	
H23C	−0.685371	0.769232	0.450510	0.130*	

### Diphenylmethyl 2-(3,5-dimethoxyphenyl)acetate (I) Atomic displacement parameters (Å^2^)

**Table:**

	*U*^11^	*U*^22^	*U*^33^	*U*^12^	*U*^13^	*U*^23^
O1	0.0603 (10)	0.0519 (9)	0.0560 (9)	−0.0077 (7)	−0.0144 (7)	0.0265 (7)
O2	0.0892 (14)	0.0892 (14)	0.0945 (14)	−0.0230 (11)	−0.0352 (11)	0.0600 (12)
O3	0.0963 (15)	0.0707 (12)	0.0643 (12)	0.0199 (11)	0.0146 (10)	0.0064 (10)
O4	0.0588 (11)	0.0948 (15)	0.0795 (13)	0.0157 (10)	−0.0064 (9)	0.0086 (11)
C1	0.0568 (14)	0.0500 (13)	0.0629 (15)	0.0052 (11)	0.0025 (11)	0.0198 (11)
C2	0.0688 (16)	0.0474 (13)	0.0523 (13)	0.0033 (11)	0.0076 (11)	0.0113 (11)
C3	0.0616 (15)	0.0540 (14)	0.0518 (13)	−0.0018 (11)	−0.0054 (11)	0.0094 (11)
C4	0.0525 (13)	0.0567 (14)	0.0604 (14)	0.0013 (11)	−0.0010 (11)	0.0156 (12)
C5	0.0613 (14)	0.0504 (13)	0.0503 (13)	0.0010 (11)	0.0037 (11)	0.0106 (10)
C6	0.0553 (13)	0.0443 (12)	0.0510 (12)	−0.0028 (10)	−0.0016 (10)	0.0178 (10)
C7	0.0621 (14)	0.0548 (14)	0.0528 (13)	−0.0013 (11)	−0.0053 (11)	0.0222 (11)
C8	0.0526 (12)	0.0509 (12)	0.0396 (11)	0.0018 (10)	−0.0014 (9)	0.0171 (9)
C9	0.0516 (12)	0.0467 (12)	0.0463 (11)	−0.0011 (9)	−0.0035 (9)	0.0211 (10)
C10	0.0530 (12)	0.0456 (12)	0.0405 (11)	0.0050 (10)	0.0025 (9)	0.0092 (9)
C11	0.0599 (15)	0.0807 (18)	0.0779 (17)	0.0186 (13)	0.0100 (13)	0.0422 (15)
C12	0.0680 (18)	0.100 (2)	0.091 (2)	0.0334 (17)	0.0089 (16)	0.0436 (18)
C13	0.0581 (16)	0.087 (2)	0.0780 (19)	0.0231 (15)	0.0132 (14)	0.0135 (16)
C14	0.0626 (17)	0.090 (2)	0.092 (2)	0.0100 (15)	0.0275 (15)	0.0309 (18)
C15	0.0651 (16)	0.0708 (17)	0.0677 (16)	0.0103 (13)	0.0143 (13)	0.0286 (13)
C16	0.0382 (10)	0.0517 (12)	0.0535 (12)	0.0034 (9)	−0.0027 (9)	0.0223 (10)
C17	0.0748 (17)	0.0646 (16)	0.0555 (14)	0.0150 (13)	0.0068 (12)	0.0235 (12)
C18	0.0821 (19)	0.089 (2)	0.0578 (16)	0.0175 (16)	0.0107 (14)	0.0180 (15)
C19	0.082 (2)	0.076 (2)	0.090 (2)	0.0287 (16)	0.0146 (17)	0.0090 (17)
C20	0.140 (3)	0.076 (2)	0.115 (3)	0.052 (2)	0.036 (2)	0.042 (2)
C21	0.106 (2)	0.0687 (18)	0.0843 (19)	0.0332 (16)	0.0236 (17)	0.0395 (16)
C22	0.130 (3)	0.094 (2)	0.0564 (17)	0.018 (2)	0.0113 (18)	0.0010 (16)
C23	0.0724 (18)	0.089 (2)	0.096 (2)	0.0257 (16)	0.0198 (16)	0.0289 (18)

### Diphenylmethyl 2-(3,5-dimethoxyphenyl)acetate (I) Geometric parameters (Å, º)

**Table:**

O1—C8	1.317 (2)	C11—H11	0.9300
O1—C9	1.460 (3)	C12—C13	1.359 (4)
O2—C8	1.197 (3)	C12—H12	0.9300
O3—C2	1.372 (3)	C13—C14	1.357 (4)
O3—C22	1.427 (4)	C13—H13	0.9300
O4—C4	1.374 (3)	C14—C15	1.385 (4)
O4—C23	1.416 (3)	C14—H14	0.9300
C1—C6	1.378 (3)	C15—H15	0.9300
C1—C2	1.390 (3)	C16—C21	1.364 (3)
C1—H1	0.9300	C16—C17	1.376 (3)
C2—C3	1.374 (3)	C17—C18	1.384 (4)
C3—C4	1.391 (3)	C17—H17	0.9300
C3—H3	0.9300	C18—C19	1.360 (4)
C4—C5	1.377 (3)	C18—H18	0.9300
C5—C6	1.392 (3)	C19—C20	1.356 (5)
C5—H5	0.9300	C19—H19	0.9300
C6—C7	1.504 (3)	C20—C21	1.381 (4)
C7—C8	1.496 (3)	C20—H20	0.9300
C7—H7A	0.9700	C21—H21	0.9300
C7—H7B	0.9700	C22—H22A	0.9600
C9—C16	1.510 (3)	C22—H22B	0.9600
C9—C10	1.511 (3)	C22—H22C	0.9600
C9—H9	0.9800	C23—H23A	0.9600
C10—C11	1.375 (3)	C23—H23B	0.9600
C10—C15	1.378 (3)	C23—H23C	0.9600
C11—C12	1.381 (4)		
			
C8—O1—C9	117.82 (16)	C13—C12—C11	120.6 (3)
C2—O3—C22	118.2 (2)	C13—C12—H12	119.7
C4—O4—C23	117.6 (2)	C11—C12—H12	119.7
C6—C1—C2	120.2 (2)	C14—C13—C12	119.6 (3)
C6—C1—H1	119.9	C14—C13—H13	120.2
C2—C1—H1	119.9	C12—C13—H13	120.2
O3—C2—C3	124.4 (2)	C13—C14—C15	120.5 (3)
O3—C2—C1	115.1 (2)	C13—C14—H14	119.8
C3—C2—C1	120.5 (2)	C15—C14—H14	119.8
C2—C3—C4	119.0 (2)	C10—C15—C14	120.4 (3)
C2—C3—H3	120.5	C10—C15—H15	119.8
C4—C3—H3	120.5	C14—C15—H15	119.8
O4—C4—C5	124.0 (2)	C21—C16—C17	118.3 (2)
O4—C4—C3	114.8 (2)	C21—C16—C9	120.0 (2)
C5—C4—C3	121.2 (2)	C17—C16—C9	121.6 (2)
C4—C5—C6	119.3 (2)	C16—C17—C18	120.8 (3)
C4—C5—H5	120.3	C16—C17—H17	119.6
C6—C5—H5	120.3	C18—C17—H17	119.6
C1—C6—C5	119.9 (2)	C19—C18—C17	119.9 (3)
C1—C6—C7	120.6 (2)	C19—C18—H18	120.0
C5—C6—C7	119.5 (2)	C17—C18—H18	120.0
C8—C7—C6	112.35 (18)	C20—C19—C18	119.7 (3)
C8—C7—H7A	109.1	C20—C19—H19	120.2
C6—C7—H7A	109.1	C18—C19—H19	120.2
C8—C7—H7B	109.1	C19—C20—C21	120.6 (3)
C6—C7—H7B	109.1	C19—C20—H20	119.7
H7A—C7—H7B	107.9	C21—C20—H20	119.7
O2—C8—O1	122.9 (2)	C16—C21—C20	120.7 (3)
O2—C8—C7	124.7 (2)	C16—C21—H21	119.7
O1—C8—C7	112.15 (18)	C20—C21—H21	119.7
O1—C9—C16	109.94 (18)	O3—C22—H22A	109.5
O1—C9—C10	107.36 (16)	O3—C22—H22B	109.5
C16—C9—C10	112.50 (17)	H22A—C22—H22B	109.5
O1—C9—H9	109.0	O3—C22—H22C	109.5
C16—C9—H9	109.0	H22A—C22—H22C	109.5
C10—C9—H9	109.0	H22B—C22—H22C	109.5
C11—C10—C15	118.3 (2)	O4—C23—H23A	109.5
C11—C10—C9	122.4 (2)	O4—C23—H23B	109.5
C15—C10—C9	119.2 (2)	H23A—C23—H23B	109.5
C10—C11—C12	120.5 (3)	O4—C23—H23C	109.5
C10—C11—H11	119.7	H23A—C23—H23C	109.5
C12—C11—H11	119.7	H23B—C23—H23C	109.5
			
C22—O3—C2—C3	8.9 (4)	O1—C9—C10—C11	−33.9 (3)
C22—O3—C2—C1	−170.5 (2)	C16—C9—C10—C11	87.2 (3)
C6—C1—C2—O3	178.9 (2)	O1—C9—C10—C15	147.8 (2)
C6—C1—C2—C3	−0.6 (4)	C16—C9—C10—C15	−91.1 (2)
O3—C2—C3—C4	−179.6 (2)	C15—C10—C11—C12	1.8 (4)
C1—C2—C3—C4	−0.1 (4)	C9—C10—C11—C12	−176.5 (2)
C23—O4—C4—C5	1.8 (4)	C10—C11—C12—C13	−0.9 (5)
C23—O4—C4—C3	−178.6 (2)	C11—C12—C13—C14	−0.4 (5)
C2—C3—C4—O4	−179.1 (2)	C12—C13—C14—C15	0.8 (5)
C2—C3—C4—C5	0.5 (4)	C11—C10—C15—C14	−1.4 (4)
O4—C4—C5—C6	179.4 (2)	C9—C10—C15—C14	176.9 (2)
C3—C4—C5—C6	−0.2 (4)	C13—C14—C15—C10	0.1 (4)
C2—C1—C6—C5	0.9 (3)	O1—C9—C16—C21	−135.5 (2)
C2—C1—C6—C7	−176.7 (2)	C10—C9—C16—C21	104.9 (3)
C4—C5—C6—C1	−0.5 (3)	O1—C9—C16—C17	47.0 (3)
C4—C5—C6—C7	177.1 (2)	C10—C9—C16—C17	−72.6 (3)
C1—C6—C7—C8	91.1 (3)	C21—C16—C17—C18	2.3 (4)
C5—C6—C7—C8	−86.6 (3)	C9—C16—C17—C18	179.8 (2)
C9—O1—C8—O2	−6.4 (3)	C16—C17—C18—C19	−1.5 (4)
C9—O1—C8—C7	178.60 (19)	C17—C18—C19—C20	−0.6 (5)
C6—C7—C8—O2	20.2 (4)	C18—C19—C20—C21	1.7 (6)
C6—C7—C8—O1	−164.9 (2)	C17—C16—C21—C20	−1.2 (4)
C8—O1—C9—C16	98.7 (2)	C9—C16—C21—C20	−178.7 (3)
C8—O1—C9—C10	−138.7 (2)	C19—C20—C21—C16	−0.8 (6)

### Diphenylmethyl 2-(3,5-dimethoxyphenyl)acetate (I) Hydrogen-bond geometry (Å, º)

**Table:**

*D*—H···*A*	*D*—H	H···*A*	*D*···*A*	*D*—H···*A*
C9—H9···O2	0.98	2.30	2.667 (3)	101
C9—H9···O2^i^	0.98	2.50	3.444 (3)	162

Symmetry code: (i) −*x*, −*y*+2, −*z*+1.

### Diphenylmethyl 2-(3,4,5-trimethoxyphenyl)acetate (II) Crystal data

**Table:**

C_24_H_24_O_5_	*F*(000) = 832
*M**_r_* = 392.43	*D*_x_ = 1.244 Mg m^−^^3^
Monoclinic, *P*2_1_/*n*	Mo *K*α radiation, λ = 0.71073 Å
*a* = 17.2290 (14) Å	Cell parameters from 5708 reflections
*b* = 5.5037 (5) Å	θ = 2.4–21.2°
*c* = 22.1140 (18) Å	µ = 0.09 mm^−^^1^
β = 92.256 (2)°	*T* = 300 K
*V* = 2095.3 (3) Å^3^	Block, colourless
*Z* = 4	0.28 × 0.09 × 0.07 mm

### Diphenylmethyl 2-(3,4,5-trimethoxyphenyl)acetate (II) Data collection

**Table:**

Bruker APEXII CCD diffractometer	2516 reflections with *I* > 2σ(*I*)
Radiation source: i-mu-s microfocus source	*R*_int_ = 0.074
φ and ω scans	θ_max_ = 28.0°, θ_min_ = 1.8°
Absorption correction: multi-scan (SADABS; Krause *et al.*, 2015)	*h* = −22→21
*T*_min_ = 0.694, *T*_max_ = 0.746	*k* = −7→7
39211 measured reflections	*l* = −22→29
5016 independent reflections	

### Diphenylmethyl 2-(3,4,5-trimethoxyphenyl)acetate (II) Refinement

**Table:**

Refinement on *F*^2^	Hydrogen site location: inferred from neighbouring sites
Least-squares matrix: full	H-atom parameters constrained
*R*[*F*^2^ > 2σ(*F*^2^)] = 0.057	*w* = 1/[σ^2^(*F*_o_^2^) + (0.0684*P*)^2^ + 0.6429*P*] where *P* = (*F*_o_^2^ + 2*F*_c_^2^)/3
*wR*(*F*^2^) = 0.176	(Δ/σ)_max_ < 0.001
*S* = 1.00	Δρ_max_ = 0.25 e Å^−^^3^
5016 reflections	Δρ_min_ = −0.18 e Å^−^^3^
263 parameters	Extinction correction: SHELXL (Sheldrick, 2015b), Fc^*^=kFc[1+0.001xFc^2^λ^3^/sin(2θ)]^-1/4^
0 restraints	Extinction coefficient: 0.0182 (19)

### Diphenylmethyl 2-(3,4,5-trimethoxyphenyl)acetate (II) Special details

**Table:**

Geometry. All esds (except the esd in the dihedral angle between two l.s. planes) are estimated using the full covariance matrix. The cell esds are taken into account individually in the estimation of esds in distances, angles and torsion angles; correlations between esds in cell parameters are only used when they are defined by crystal symmetry. An approximate (isotropic) treatment of cell esds is used for estimating esds involving l.s. planes.

### Diphenylmethyl 2-(3,4,5-trimethoxyphenyl)acetate (II) Fractional atomic coordinates and isotropic or equivalent isotropic displacement parameters (Å^2^)

**Table:**

	*x*	*y*	*z*	*U*_iso_*/*U*_eq_	
O1	0.61673 (10)	0.1274 (3)	0.05557 (6)	0.0636 (5)	
O2	0.58110 (11)	−0.1961 (3)	0.00128 (7)	0.0774 (5)	
O3	0.85376 (10)	0.5998 (4)	−0.03155 (8)	0.0798 (6)	
O4	0.82226 (11)	−0.0249 (4)	−0.17236 (8)	0.0859 (6)	
O5	0.90848 (10)	0.3195 (4)	−0.11910 (8)	0.0862 (6)	
C1	0.72746 (12)	0.4012 (4)	−0.04036 (9)	0.0546 (6)	
H1	0.706890	0.501478	−0.011177	0.066*	
C2	0.80384 (13)	0.4295 (5)	−0.05603 (10)	0.0565 (6)	
C3	0.83399 (13)	0.2815 (5)	−0.10043 (10)	0.0610 (7)	
C4	0.78760 (14)	0.1072 (5)	−0.12858 (10)	0.0613 (6)	
C5	0.71122 (13)	0.0768 (4)	−0.11256 (9)	0.0575 (6)	
H5	0.680153	−0.040984	−0.131508	0.069*	
C6	0.68155 (12)	0.2237 (4)	−0.06806 (9)	0.0507 (6)	
C7	0.59898 (12)	0.1867 (5)	−0.04865 (9)	0.0554 (6)	
H7A	0.577106	0.341746	−0.037250	0.066*	
H7B	0.567537	0.122130	−0.082264	0.066*	
C8	0.59723 (12)	0.0140 (5)	0.00406 (9)	0.0494 (5)	
C9	0.61902 (14)	−0.0116 (4)	0.11119 (9)	0.0580 (6)	
H9	0.603757	−0.179139	0.101590	0.070*	
C10	0.70113 (14)	−0.0137 (4)	0.13712 (9)	0.0573 (6)	
C11	0.75246 (16)	0.1724 (5)	0.12627 (12)	0.0738 (7)	
H11	0.736653	0.302650	0.101974	0.089*	
C12	0.82733 (18)	0.1664 (6)	0.15134 (14)	0.0893 (9)	
H12	0.861775	0.291515	0.143454	0.107*	
C13	0.85089 (19)	−0.0237 (7)	0.18780 (14)	0.0899 (10)	
H13	0.901014	−0.026577	0.205006	0.108*	
C14	0.8005 (2)	−0.2082 (7)	0.19871 (13)	0.0912 (10)	
H14	0.816513	−0.336963	0.223414	0.109*	
C15	0.72640 (18)	−0.2053 (5)	0.17351 (12)	0.0768 (8)	
H15	0.692821	−0.333234	0.180921	0.092*	
C16	0.56005 (13)	0.0950 (4)	0.15319 (9)	0.0559 (6)	
C17	0.54475 (15)	−0.0247 (5)	0.20629 (11)	0.0732 (7)	
H17	0.571537	−0.166755	0.216249	0.088*	
C18	0.49038 (16)	0.0633 (6)	0.24472 (12)	0.0839 (9)	
H18	0.480967	−0.019706	0.280326	0.101*	
C19	0.45026 (16)	0.2707 (6)	0.23110 (13)	0.0812 (8)	
H19	0.413734	0.330233	0.257196	0.097*	
C20	0.46445 (17)	0.3897 (6)	0.17866 (14)	0.0851 (8)	
H20	0.436827	0.530448	0.168889	0.102*	
C21	0.51904 (16)	0.3054 (5)	0.13979 (12)	0.0711 (7)	
H21	0.528292	0.390302	0.104434	0.085*	
C22	0.82561 (16)	0.7587 (5)	0.01338 (12)	0.0789 (8)	
H22A	0.866226	0.868081	0.026580	0.118*	
H22B	0.782408	0.850000	−0.003331	0.118*	
H22C	0.809195	0.665036	0.047205	0.118*	
C23	0.78071 (19)	−0.2196 (5)	−0.19993 (13)	0.0898 (9)	
H23A	0.812069	−0.295090	−0.229561	0.135*	
H23B	0.768119	−0.336789	−0.169695	0.135*	
H23C	0.733721	−0.159175	−0.219272	0.135*	
C24	0.96593 (19)	0.1834 (11)	−0.08755 (19)	0.179 (2)	
H24A	1.015823	0.219968	−0.103154	0.268*	
H24B	0.966039	0.224012	−0.045331	0.268*	
H24C	0.955212	0.013311	−0.092616	0.268*	

### Diphenylmethyl 2-(3,4,5-trimethoxyphenyl)acetate (II) Atomic displacement parameters (Å^2^)

**Table:**

	*U*^11^	*U*^22^	*U*^33^	*U*^12^	*U*^13^	*U*^23^
O1	0.0941 (12)	0.0551 (10)	0.0410 (8)	−0.0129 (9)	−0.0040 (8)	0.0041 (7)
O2	0.1106 (14)	0.0619 (12)	0.0595 (10)	−0.0110 (11)	−0.0007 (9)	−0.0093 (9)
O3	0.0633 (11)	0.0989 (14)	0.0781 (12)	−0.0191 (10)	0.0144 (9)	−0.0194 (11)
O4	0.0838 (12)	0.1052 (15)	0.0708 (11)	−0.0005 (11)	0.0304 (9)	−0.0254 (11)
O5	0.0574 (10)	0.1270 (17)	0.0761 (12)	−0.0066 (11)	0.0265 (9)	−0.0072 (11)
C1	0.0535 (13)	0.0660 (15)	0.0450 (12)	0.0037 (12)	0.0079 (10)	0.0004 (11)
C2	0.0489 (13)	0.0717 (16)	0.0492 (12)	−0.0056 (12)	0.0053 (10)	0.0017 (12)
C3	0.0485 (13)	0.0861 (18)	0.0494 (13)	0.0002 (13)	0.0144 (10)	0.0036 (13)
C4	0.0641 (15)	0.0761 (17)	0.0447 (12)	0.0082 (13)	0.0145 (11)	−0.0032 (12)
C5	0.0586 (14)	0.0704 (16)	0.0439 (12)	−0.0009 (12)	0.0068 (10)	−0.0011 (11)
C6	0.0466 (12)	0.0655 (15)	0.0402 (11)	0.0031 (11)	0.0045 (9)	0.0066 (11)
C7	0.0457 (12)	0.0795 (16)	0.0409 (11)	0.0010 (11)	0.0004 (9)	0.0031 (11)
C8	0.0405 (11)	0.0641 (16)	0.0441 (12)	0.0016 (11)	0.0061 (9)	−0.0060 (12)
C9	0.0848 (17)	0.0471 (13)	0.0419 (12)	−0.0093 (12)	0.0001 (11)	0.0049 (10)
C10	0.0752 (16)	0.0541 (14)	0.0428 (12)	0.0080 (13)	0.0068 (11)	−0.0048 (11)
C11	0.0811 (19)	0.0673 (18)	0.0720 (17)	0.0013 (15)	−0.0073 (14)	0.0010 (14)
C12	0.078 (2)	0.093 (2)	0.096 (2)	0.0014 (17)	−0.0086 (17)	−0.0139 (19)
C13	0.079 (2)	0.113 (3)	0.0759 (19)	0.034 (2)	−0.0136 (16)	−0.029 (2)
C14	0.101 (2)	0.101 (3)	0.0714 (18)	0.041 (2)	−0.0015 (17)	0.0027 (18)
C15	0.095 (2)	0.0737 (19)	0.0624 (15)	0.0222 (16)	0.0108 (15)	0.0083 (14)
C16	0.0619 (14)	0.0592 (15)	0.0461 (12)	−0.0111 (12)	−0.0039 (10)	0.0006 (11)
C17	0.0686 (16)	0.0880 (19)	0.0632 (15)	−0.0053 (15)	0.0052 (13)	0.0181 (15)
C18	0.0687 (17)	0.119 (3)	0.0645 (17)	−0.0058 (18)	0.0144 (14)	0.0183 (17)
C19	0.0638 (17)	0.107 (2)	0.0728 (18)	−0.0014 (17)	0.0071 (14)	−0.0101 (18)
C20	0.085 (2)	0.089 (2)	0.082 (2)	0.0120 (17)	0.0052 (16)	−0.0068 (17)
C21	0.0866 (18)	0.0663 (17)	0.0603 (15)	−0.0007 (15)	0.0017 (14)	0.0045 (13)
C22	0.0846 (18)	0.085 (2)	0.0675 (16)	−0.0140 (16)	0.0031 (14)	−0.0134 (15)
C23	0.121 (2)	0.080 (2)	0.0706 (17)	0.0092 (19)	0.0276 (17)	−0.0136 (16)
C24	0.060 (2)	0.334 (7)	0.144 (4)	0.053 (3)	0.020 (2)	0.058 (4)

### Diphenylmethyl 2-(3,4,5-trimethoxyphenyl)acetate (II) Geometric parameters (Å, º)

**Table:**

O1—C8	1.330 (3)	C12—C13	1.372 (4)
O1—C9	1.448 (2)	C12—H12	0.9300
O2—C8	1.191 (3)	C13—C14	1.363 (4)
O3—C2	1.369 (3)	C13—H13	0.9300
O3—C22	1.423 (3)	C14—C15	1.373 (4)
O4—C4	1.367 (3)	C14—H14	0.9300
O4—C23	1.414 (3)	C15—H15	0.9300
O5—C3	1.379 (3)	C16—C21	1.382 (3)
O5—C24	1.405 (4)	C16—C17	1.381 (3)
C1—C2	1.383 (3)	C17—C18	1.377 (4)
C1—C6	1.384 (3)	C17—H17	0.9300
C1—H1	0.9300	C18—C19	1.362 (4)
C2—C3	1.392 (3)	C18—H18	0.9300
C3—C4	1.381 (3)	C19—C20	1.362 (4)
C4—C5	1.386 (3)	C19—H19	0.9300
C5—C6	1.387 (3)	C20—C21	1.380 (4)
C5—H5	0.9300	C20—H20	0.9300
C6—C7	1.516 (3)	C21—H21	0.9300
C7—C8	1.505 (3)	C22—H22A	0.9600
C7—H7A	0.9700	C22—H22B	0.9600
C7—H7B	0.9700	C22—H22C	0.9600
C9—C10	1.506 (3)	C23—H23A	0.9600
C9—C16	1.521 (3)	C23—H23B	0.9600
C9—H9	0.9800	C23—H23C	0.9600
C10—C11	1.381 (4)	C24—H24A	0.9600
C10—C15	1.386 (3)	C24—H24B	0.9600
C11—C12	1.384 (4)	C24—H24C	0.9600
C11—H11	0.9300		
			
C8—O1—C9	118.49 (18)	C14—C13—C12	119.7 (3)
C2—O3—C22	118.07 (19)	C14—C13—H13	120.1
C4—O4—C23	118.7 (2)	C12—C13—H13	120.1
C3—O5—C24	114.6 (2)	C13—C14—C15	120.5 (3)
C2—C1—C6	120.1 (2)	C13—C14—H14	119.7
C2—C1—H1	119.9	C15—C14—H14	119.7
C6—C1—H1	119.9	C14—C15—C10	120.7 (3)
O3—C2—C1	124.6 (2)	C14—C15—H15	119.7
O3—C2—C3	115.68 (19)	C10—C15—H15	119.7
C1—C2—C3	119.7 (2)	C21—C16—C17	118.0 (2)
C4—C3—O5	119.9 (2)	C21—C16—C9	122.7 (2)
C4—C3—C2	119.9 (2)	C17—C16—C9	119.3 (2)
O5—C3—C2	120.0 (2)	C18—C17—C16	120.9 (3)
O4—C4—C3	115.3 (2)	C18—C17—H17	119.5
O4—C4—C5	124.2 (2)	C16—C17—H17	119.5
C3—C4—C5	120.5 (2)	C19—C18—C17	120.6 (3)
C4—C5—C6	119.4 (2)	C19—C18—H18	119.7
C4—C5—H5	120.3	C17—C18—H18	119.7
C6—C5—H5	120.3	C20—C19—C18	119.0 (3)
C1—C6—C5	120.3 (2)	C20—C19—H19	120.5
C1—C6—C7	119.7 (2)	C18—C19—H19	120.5
C5—C6—C7	119.9 (2)	C19—C20—C21	121.2 (3)
C8—C7—C6	110.59 (17)	C19—C20—H20	119.4
C8—C7—H7A	109.5	C21—C20—H20	119.4
C6—C7—H7A	109.5	C20—C21—C16	120.2 (3)
C8—C7—H7B	109.5	C20—C21—H21	119.9
C6—C7—H7B	109.5	C16—C21—H21	119.9
H7A—C7—H7B	108.1	O3—C22—H22A	109.5
O2—C8—O1	123.4 (2)	O3—C22—H22B	109.5
O2—C8—C7	125.8 (2)	H22A—C22—H22B	109.5
O1—C8—C7	110.8 (2)	O3—C22—H22C	109.5
O1—C9—C10	108.75 (18)	H22A—C22—H22C	109.5
O1—C9—C16	108.58 (19)	H22B—C22—H22C	109.5
C10—C9—C16	114.24 (17)	O4—C23—H23A	109.5
O1—C9—H9	108.4	O4—C23—H23B	109.5
C10—C9—H9	108.4	H23A—C23—H23B	109.5
C16—C9—H9	108.4	O4—C23—H23C	109.5
C11—C10—C15	118.5 (2)	H23A—C23—H23C	109.5
C11—C10—C9	121.8 (2)	H23B—C23—H23C	109.5
C15—C10—C9	119.7 (2)	O5—C24—H24A	109.5
C10—C11—C12	120.4 (3)	O5—C24—H24B	109.5
C10—C11—H11	119.8	H24A—C24—H24B	109.5
C12—C11—H11	119.8	O5—C24—H24C	109.5
C13—C12—C11	120.2 (3)	H24A—C24—H24C	109.5
C13—C12—H12	119.9	H24B—C24—H24C	109.5
C11—C12—H12	119.9		
			
C22—O3—C2—C1	0.3 (3)	C6—C7—C8—O1	79.9 (2)
C22—O3—C2—C3	179.0 (2)	C8—O1—C9—C10	116.5 (2)
C6—C1—C2—O3	179.7 (2)	C8—O1—C9—C16	−118.7 (2)
C6—C1—C2—C3	1.0 (3)	O1—C9—C10—C11	27.6 (3)
C24—O5—C3—C4	−92.2 (4)	C16—C9—C10—C11	−93.8 (3)
C24—O5—C3—C2	92.1 (4)	O1—C9—C10—C15	−153.1 (2)
O3—C2—C3—C4	−178.8 (2)	C16—C9—C10—C15	85.5 (3)
C1—C2—C3—C4	0.0 (4)	C15—C10—C11—C12	0.0 (4)
O3—C2—C3—O5	−3.1 (3)	C9—C10—C11—C12	179.3 (2)
C1—C2—C3—O5	175.7 (2)	C10—C11—C12—C13	−0.7 (4)
C23—O4—C4—C3	175.0 (2)	C11—C12—C13—C14	0.8 (4)
C23—O4—C4—C5	−5.7 (4)	C12—C13—C14—C15	0.0 (5)
O5—C3—C4—O4	2.9 (3)	C13—C14—C15—C10	−0.8 (4)
C2—C3—C4—O4	178.7 (2)	C11—C10—C15—C14	0.8 (4)
O5—C3—C4—C5	−176.3 (2)	C9—C10—C15—C14	−178.5 (2)
C2—C3—C4—C5	−0.6 (4)	O1—C9—C16—C21	−6.0 (3)
O4—C4—C5—C6	−179.0 (2)	C10—C9—C16—C21	115.6 (2)
C3—C4—C5—C6	0.2 (3)	O1—C9—C16—C17	172.25 (19)
C2—C1—C6—C5	−1.4 (3)	C10—C9—C16—C17	−66.2 (3)
C2—C1—C6—C7	177.4 (2)	C21—C16—C17—C18	−0.1 (4)
C4—C5—C6—C1	0.8 (3)	C9—C16—C17—C18	−178.4 (2)
C4—C5—C6—C7	−178.0 (2)	C16—C17—C18—C19	0.1 (4)
C1—C6—C7—C8	−87.0 (3)	C17—C18—C19—C20	0.3 (4)
C5—C6—C7—C8	91.8 (2)	C18—C19—C20—C21	−0.7 (4)
C9—O1—C8—O2	0.4 (3)	C19—C20—C21—C16	0.7 (4)
C9—O1—C8—C7	−179.34 (18)	C17—C16—C21—C20	−0.3 (4)
C6—C7—C8—O2	−99.8 (3)	C9—C16—C21—C20	177.9 (2)

### Diphenylmethyl 2-(3,4,5-trimethoxyphenyl)acetate (II) Hydrogen-bond geometry (Å, º)

**Table:**

*D*—H···*A*	*D*—H	H···*A*	*D*···*A*	*D*—H···*A*
C9—H9···O2	0.98	2.24	2.691 (3)	107
C21—H21···O1	0.93	2.39	2.740 (3)	102
C19—H19···O4^i^	0.93	2.50	3.427 (3)	175

Symmetry code: (i) *x*−1/2, −*y*+1/2, *z*+1/2.
